# The novel Leal-polynomials for the multi-expansive approximation of nonlinear differential equations

**DOI:** 10.1016/j.heliyon.2020.e03695

**Published:** 2020-04-14

**Authors:** Hector Vazquez-Leal, Mario Alberto Sandoval-Hernandez, Uriel Filobello-Nino, Jesus Huerta-Chua

**Affiliations:** a Facultad de Instrumentación Electrónica, Universidad Veracruzana, Cto. Gonzalo Aguirre Beltrán S/N, Xalapa, Veracruz, 91000, Mexico; b Consejo Veracruzano de Investigación Científica y Desarrollo Tecnológico (COVEICYDET), Av Rafael Murillo Vidal No. 1735, Cuauhtemoc, 91069, Xalapa, Veracruz, Mexico; c Universidad de Xalapa, Escuela de Ingeniería, Carretera Xalapa-Veracruz Km 2 No. 341, 91190, Xalapa, Veracruz, Mexico; d DGETI-SEP CB190, Av. 15, esq. calle 11. Col. Venustiano Carranza Boca del Río, Ver., C.P. 94297, Mexico; e Instituto Tecnológico Superior de Poza Rica, Tecnológico Nacional de México, Luis Donaldo Colosio Murrieta S/N, Arroyo del Maíz, C.P. 93230 Poza Rica, Veracruz, Mexico

**Keywords:** Mathematics, Differential equations, Semi-analytic approximations, Taylor series, Multiple expansions

## Abstract

This work presents the novel Leal-polynomials (LP) for the approximation of nonlinear differential equations of different kind. The main characteristic of LPs is that they satisfy multiple expansion points and its derivatives as a mechanism to replicate behaviour of the nonlinear problem, giving more accuracy within the region of interest. Therefore, the main contribution of this work is that LP satisfies the successive derivatives in some specific points, resulting more accurate polynomials than Taylor expansion does for the same degree of their respective polynomials. Such characteristic makes of LPs a handy and powerful tool to approximate different kind of differential equations including: singular problems, initial condition and boundary-valued problems, equations with discontinuities, coupled differential equations, high-order equations, among others. Additionally, we show how the process to obtain the polynomials is straightforward and simple to implement; generating a compact, and easy to compute, expression. Even more, we present the process to approximate Gelfand's equation, an equation of an isothermal reaction, a model for chronic myelogenous leukemia, Thomas-Fermi equation, and a high order nonlinear differential equations with discontinuities getting, as result, accurate, fast and compact approximate solutions. In addition, we present the computational convergence and error studies for LPs resulting convergent polynomials and error tendency to zero as the order of LPs increases for all study cases. Finally, a study of CPU time shows that LPs require a few nano-seconds to be evaluated, which makes them suitable for intensive computing applications.

## 1 Introduction

All kinds of nonlinear differential equations emanate when modelling different phenomenon of sciences and engineering without knowing an exact solution. Unlike the theory of linear ordinary differential equations, relatively little is known about the general nature of nonlinear ODES; specially when explicit solutions cannot be obtained. The reason to study these equations is that many physical systems (and equations that describe them) are of nonlinear nature. Although there exists the qualitative theory of nonlinear ODES, which was discovered by Henri Poincaré in 1880, it just provides the general behaviour of the solutions, in particular, only describes the asymptotic character of them. From the aforementioned, it is clear the importance of searching for methods which provide, at least, analytical approximate solutions for nonlinear ODES in the whole domain of the problem to solve. In addition, another tool to understand the behaviour of solutions, indirectly, is through the application of numerical methods. Nonetheless, such algorithms exhibit several issues like: false states of equilibrium, oscillations, numerical instabilities, among others [1], [2]. Therefore, the obtained numerical solution may not represent the desired result [3], [4]. Hence, a line of research is focused to obtain solutions in the form of analytical approximations like: enhanced power series method [5], power series extender method [6], [7], modified Taylor series method [8], direct Padé method [9], [10], rational homotopy perturbation method [11], generalized homotopy method [12], [13], homotopy analysis method [2], [14], homotopy perturbation method [15], [16], [17], among others. On one hand, all above mentioned methods may provide approximate solutions in terms of polynomials; nevertheless, users require specialized knowledge of mathematics to apply such methods. On the other hand, we propose a novel method that is capable to produce accurate multi-expansive polynomials requiring just basic knowledge of mathematics. Such multi-expansive expressions will be denominated as Leal-polynomials (LP) for the rest of this work.

The Leal-polynomials are applied to obtain accurate approximations of a wide spectrum of nonlinear differential equations. Such polynomials are constructed to satisfy the boundaries and their respective derivatives depending on the selected order for the approximation. In contrast with Taylor expansion, LPs can expand simultaneously in multiple points, providing accurate polynomials. Also, as LP increases its order, the approximation's accuracy improves within the inner region of the expansion points. Even-more, the missing derivatives required by LP are obtained using either a numerical method or the least squares method regarding the intrinsic parameters of the nonlinear problem. Hence, we will present as study cases some important nonlinear problems in physics and engineering: approximate Gelfand's equation [18], [19], [20], an equation of an isothermal reaction [21], a model for chronic myelogenous leukemia [22], Thomas-Fermi equation [23], [24], and a high order nonlinear differential equations with discontinuities [25], [26], [27], [28], [29]; resulting in accurate approximations with a wide domain of convergence.

The rest of this work is organized as follows. Section 2 introduces the basic procedure to obtain the Leal-polynomials. Next, for Section 3, we present a brief introduction to the least squares method. Cases study are presented in Section 4. Later, in Section 5, we introduce studies of computing convergence and error; while, Section 6 presents a study of CPU time consumption of LPs. Next, Section 7 provides a detailed discussion of the results. Finally, conclusions of the work are exposed in Section 8.

## 2 The basic procedure to obtain the Leal-polynomials

Let us assume we have the following nonlinear differential equation:

(1) L(u)+N(u)−f(x)=0,wherex∈Ω,

having as boundary condition

(2) $$B(u,\frac{\partial u}{\partial \eta })=0,\;\text{where}\;x\in \Gamma ,$$

where *L* and *N*, are a linear and a non-linear operator, respectively; f(x) is a known analytic function, *B* is a boundary operator, Γ is the boundary of domain Ω, and ∂u/∂η denotes differentiation along the normal drawn outwards from Ω. In fact, the proposed LPs can be applied to initial conditions problems or boundary-valued problems.

First, we define Leal-polynomials as

(3) $$L(x)=\sum_{i=0}^{r}c_{i}x^{i},$$

(4) $$r=q_{a}+q_{b}+\ldots +M-1,$$

where $\left[q_{a},q_{b},\ldots \right]$ are the orders for *M*-expansion points, and *c* are the unknown coefficients to be determined. The process is a straightforward scheme that can be summarized as:

1. First, we build the LP-equations for each expansion point (EP)
  (5) $$L(a)=X_{0},\;L(b)=Y_{0},\;\ldots L^{\prime }(a)=X_{1},\;L^{\prime }(b)=Y_{1},\;\ldots \vdots \;\;\;\vdots \;\;\;\;\vdots L^{\left(q_{a}\right)}(a)=X_{q_{a}},\;L^{\left(q_{b}\right)}(b)=Y_{q_{b}},\;\ldots$$
  It is important to remark two aspects: the expansion points [a,b,…] are selected to enlarge the domain of convergence or to increase the accuracy over a finite domain, and the expansions points must - at least - include the boundary-value points of (1).
2. Linear equations system (5) is solved to obtain the *c* constants.
3. LP (3) is constructed using the *c* constants from the last step. The Algorithm 1 shows how to implement the last steps. In addition, the LP can be either numeric or symbolic depending on the implementation.

**Algorithm 1:**
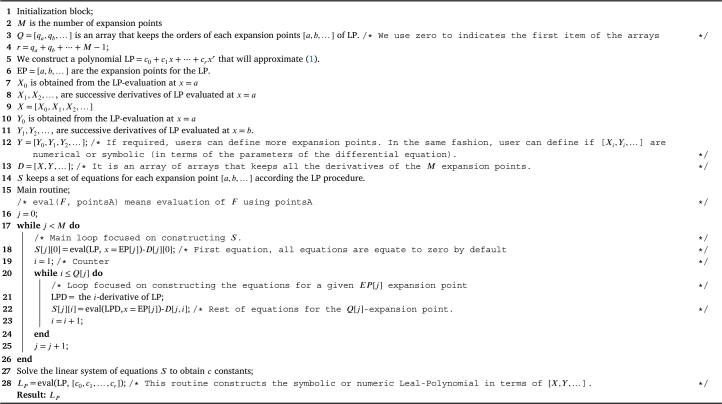
General procedure to obtain a numeric or symbolic Leal-Polynomial.

Each particular problem provides a set of initial conditions or boundary conditions that belong to the sets [X,Y,…]. Nonetheless, there is a certain number of unknowns $\left[X_{i},Y_{i},\ldots \right]$ that must be determined to initialize Taylor series method; there are two ways to solve this issue: a) the values will be obtained using a suitable numerical method, or b) such unknowns can be modelled in terms of the intrinsic parameters of the problem using the least square method (see next section for details) to construct the model. In addition, it is important to remark that the next derivatives are obtained using the well-known method of Taylor series for differential equations [8]. To facilitate the application of LP, Appendix A and Appendix C present the Maple 15 procedures to automatically generate the symbolic Leal-polynomials of two expansion points of the same order, and three expansion points of different order using Algorithm 1.

## 3 A brief introduction to the least squares method

Let ($x_{0},y_{0}$), ($x_{1},y_{1}$), ($x_{2},x_{3}$), ⋯, ($x_{i},y_{i}$), the coordinates of a data set, such that i=0,1,2,…,n, and the adjustment curve is $y=f(x,a_{0},a_{1},a_{2},\cdots ,a_{j})$, where $\left[a_{0},a_{1},\ldots ,a_{j}\right]$ are adjustment constants. The least squares approximations [30], [31], [32], [33], [34], [35] attempts to minimize the sum of squares from the vertical distances of $y_{i}$ values to the ideal model f(x) and obtain the model function $S(a_{0},a_{1},a_{2},\cdots ,a_{j})$, which minimizes the square error defined by:

(6) $$S(a_{0},a_{1},a_{2},\cdots ,a_{j})=\sum_{i=1}^{n}{\left((y_{i})-f(x_{i},a_{0},a_{1},a_{2},\cdots ,a_{j})\right)}^{2}.$$

Therefore, (6) is designed to minimize the error with respect to the sample set. To do this, partial derivatives are defined with respect to every adjustment constants, creating a system of nonlinear equations given as:

(7) $$\frac{\partial S}{\partial a_{0}}=\frac{\partial }{\partial a_{0}}(\sum_{i=1}^{n}{\left((y_{i})-f(x_{i},a_{0},a_{1},a_{2},\cdots ,a_{j})\right)}^{2})=0\;\frac{\partial S}{\partial a_{1}}=\frac{\partial }{\partial a_{1}}(\sum_{i=1}^{n}{\left((y_{i})-f(x_{i},a_{0},a_{1},a_{2},\cdots ,a_{j})\right)}^{2})=0\;\frac{\partial S}{\partial a_{2}}=\frac{\partial }{\partial a_{2}}(\sum_{i=1}^{n}{\left((y_{i})-f(x_{i},a_{0},a_{1},a_{2},\cdots ,a_{j})\right)}^{2})=0\vdots \frac{\partial S}{\partial a_{n}}=\frac{\partial S}{\partial a_{n}}(\sum_{i=1}^{n}{\left((y_{i})-f(x_{i},a_{0},a_{1},a_{2},\cdots ,a_{j})\right)}^{2})=0.$$

**Figure 1:**
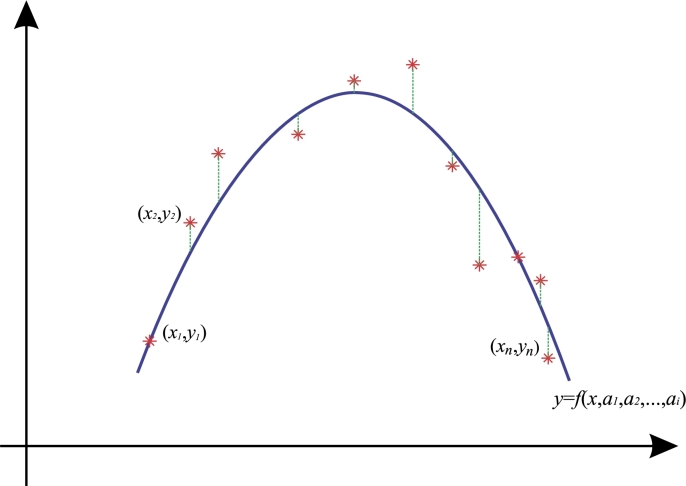
Least squares method.

By solving system (7) for adjustment constants, it is possible to obtain a model that provides the best fit to the data set. This method allows to create a continuous function in the space with a minimized error with respect to the samples [31]. In consequence, it is possible to test various models and select the one that provides the best fit. Fig. 1 depicts the methodology, where the asterisk symbol represents the data set. For every datum a value $\left(x_{i},y_{i}\right)$ is given (where i=1,2,3,…). It is possible to obtain the best function that fits the data set, represented by the blue line.

## 4 Study cases

### 4.1 Bratu's problem in 1D

In this example we will find an analytical approximate solution for Bratu's equation (that is, the Liouville-Bratu's-Gelfand equation for the one-dimensional case). This equation is important because it has many applications in science and engineering to model physical and chemical systems. The Bratu's model describes the fuel ignition model in thermal combustion theory, chemical reaction, nano-technology, radiative heat transfer, and also is helpful in the Chandrasekhar model for the expansion of the universe, among others [18], [36]. From the aforementioned, it is possible to understand the amount of research work that has been dedicated to this problem until now. Thus, the Bratu's problem [18] is given by

(8) $$y^{\prime \prime }(x)+\Phi \exp(y(x))=0,\;y(0)=0,\;y(1)=0,\;\Phi >0$$

where prime denotes differentiation with respect to *x*, Φ is the intrinsic parameter of Bratu's problem.

To calculate the Leal-polynomial, it will be necessary to approximate $\overset{˜}{y\prime }(0)$ and $\overset{˜}{y\prime }(1)$ for (8). We will express both derivatives in terms of Φ. The exact solution was calculated numerically using a scheme based on trapezoid combined with Richardson extrapolation from the built-in numerical routines provided by Maple 15. Thence, we apply the square residual error (Polynomial Fit command from Maple) in the range of Φ=[0,3.45], with intervals of ΔΦ=0.05 to polynomials of 12-th degree, resulting

(9) $$\overset{˜}{y\prime }(0)=0.01598501841+0.00161545607\Phi +4.95912118309\Phi^{2}-22.737289661\Phi^{3}+58.775902709\Phi^{4}-93.378600623\Phi^{5}+96.3303477826\Phi^{6}-66.4025908369\Phi^{7}+30.83469911315\Phi^{8}-9.520125256057\Phi^{9}+1.872596434172\Phi^{10}-0.212289669801\Phi^{11}+0.010554445035\Phi^{12},$$

and

(10) $$\overset{˜}{y\prime }(1)=-0.0159850184098-0.00161545607\Phi -4.959121183091\Phi^{2}+22.7372896610\Phi^{3}-58.7759027087\Phi^{4}+93.3786006231\Phi^{5}-96.33034778259\Phi^{6}+66.40259083691\Phi^{7}-30.83469911315\Phi^{8}+9.520125256057\Phi^{9}-1.872596434172\Phi^{10}+0.212289669801\Phi^{11}-0.010554445035\Phi^{12}.$$

In order to obtain the superior order derivatives for the left frontier (x=0), we apply the Taylor series method to (8), assuming the problem is now of initial conditions as follows

(11) $$X_{0}=y(0)=0,\;X_{1}=\overset{˜}{y\prime }(0),$$

resulting the next superior derivatives

(12) $$X_{2}=-\Phi \exp(X_{0}),X_{3}=-\Phi X_{1}\exp(X_{0}),X_{4}=(\Phi \exp(X_{0})-X_{1}^{2})\Phi \exp(X_{0}),\vdots$$

Now, we repeat the same procedure for the right boundary, resulting

(13) $$Y_{0}=y(1)=0,\;Y_{1}=\overset{˜}{y^{\prime }}(1),$$

and

(14) $$Y_{2}=-\Phi \exp(Y_{1}),Y_{3}=-\Phi Y_{1}\exp(Y_{1}),Y_{4}=(\Phi \exp(Y_{1})-Y_{1}^{2})\Phi \exp(Y_{1}),\vdots$$

Using the derivatives mentioned above and (B.5) (from Appendix B), we can obtain a symbolic Leal-polynomial of order [4,4]. For instance, we use Φ=3.4, resulting:

(15) $$\overset{˜}{y}(x)=-2.353570116956\times 10^{-9}x^{9}+1.02695234577x^{8}-4.107809383098x^{7}+5.013344070797x^{6}-0.6626993715473x^{5}-0.9448762677414x^{4}-1.798192792219x^{3}-1.70x^{2}+3.173281398x.$$

**Figure 2:**
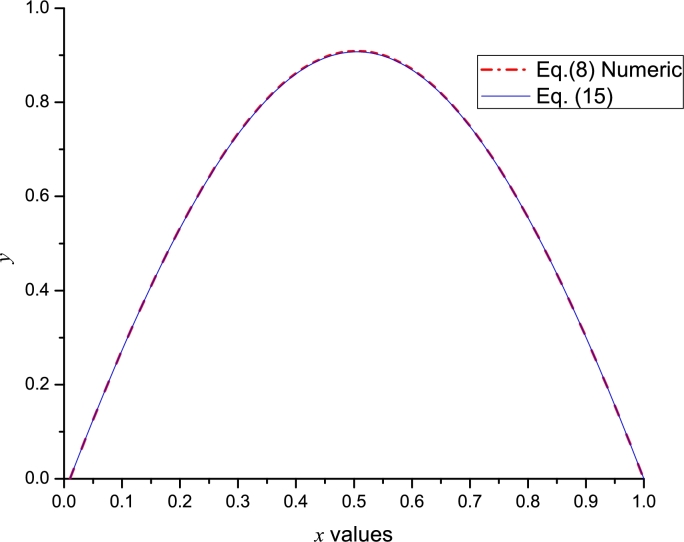
Comparison between numerical solution of (8) and (15) using Φ = 3.4.

Fig. 2 shows a good agreement between (15) and the numerical solution of Gelfand equation. With the aim to perform a comparison with Taylor method; we numerically calculated (considering Φ=3.4 and using *dsolve* command from Maple) the missing derivatives: $\overset{˜}{y^{\prime }}(0)=3.17473085207040$ and $\overset{˜}{y\prime }(1)=-3.17473085207040$, for Bratu's problem in order to construct the Taylor series ${\overset{˜}{y}}_{L}(x)$ (employing (11) and (12)) and ${\overset{˜}{y}}_{R}(x)$ (employing (13) and (14)), resulting

(16) $${\overset{˜}{y}}_{L}(x)=3.173281398136x-1.70x^{2}-1.798192792278x^{3}-0.944876267833x^{4}+0.317406667211x^{5}+1.081238617931x^{6}+0.847127841554x^{7}-0.083371054338x^{8}-7.85x^{9},\;{\overset{˜}{y}}_{R}(x)=-3.173281398062x+3.173281398062-1.700{\left(x-1\right)}^{2}+1.798192792234{\left(x-1\right)}^{3}-0.944876267765{\left(x-1\right)}^{4}-0.317406667246{\left(x-1\right)}^{5}+1.081238617892{\left(x-1\right)}^{6}-0.847127841541{\left(x-1\right)}^{7}-0.083219378380{\left(x-1\right)}^{8}+1.760{\left(x-1\right)}^{9}.$$

**Figure 3:**
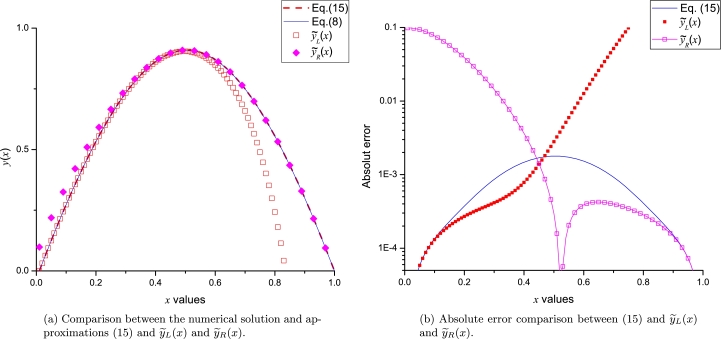
Comparison between LP (15) and Taylor series (16).

It is important to note that, on one side, (16) exhibits poor accuracy on the opposite frontier to their respective expansion points; on the other side, LP satisfies, as expected, both boundaries. This result represents a notable advantage of LP over the classical Taylor expansions, considering that two expansion points will reduce the approximation error in the inner zone between *a* and *b* frontiers (see Fig. 3a). Fig. 3b presents a comparison of absolute error between LP and Taylor series obtained for both study cases. LP exhibits high accuracy in the vicinity of the boundaries, reaching, as expected, maximum error of $\approx 10^{-3}$ at x=0.5 (right in the middle of the boundaries). On the other hand, both ${\overset{˜}{y}}_{L}(x)$ and ${\overset{˜}{y}}_{R}$ exhibit good accuracy at the vicinity of their respective expansion points, and a poor performance on the opposite boundary.

### 4.2 Nonlinear model of diffusion and reaction in porous catalysts

This example studies the nonlinear ODE that describes the diffusion and reaction in porous catalysts. The following nonlinear problem models the steady one-dimensional case, where the reaction rate depends non-linearly on concentration; so that the system can be conceived as a solid material with pores through which reactants and products diffuse [21], [37]. Next, we will get an analytical approximate solution for this problem, which properly describes the phenomenon under study. Obtained results will be valuable for chemical engineers.

An isothermal reaction that is *n*-th order and irreversible in planar geometry [21], is given by

(17) $$z^{\prime \prime }(x)-\Phi^{2}z{\left(x\right)}^{n}=0$$

with boundary conditions

(18) $$z^{\prime }(0)=0,\;z(1)=1,$$

here prime denotes differentiation with respect to *x*, Φ is Thiele modulus and *n* is the order of reaction.

Next, we repeat the same procedure from the first case study to obtain $\overset{˜}{z}(0)$ and $\overset{˜}{z\prime }(1)$ in the range of Φ=[0,7] (using ΔΦ=0.1), resulting

(19) $$\overset{˜}{z}(0)=0.996793340403603+0.0574461330628034\Phi -0.849446625608658\Phi^{2}+0.976929909334565\Phi^{3}-0.636865077147846\Phi^{4}+0.272392692810808\Phi^{5}-0.07958950734964\Phi^{6}+0.015992137070257\Phi^{7}-0.0021731758472172\Phi^{8}+0.0001907178651630\Phi^{9}-0.000009751513556\Phi^{10},$$

and

(20) $$\overset{˜}{z^{\prime }}(1)=0.005287296431409-0.093240918174492\Phi +1.55727232236114\Phi^{2}-1.5321871725448\Phi^{3}+0.946027701324267\Phi^{4}-0.392653692072043\Phi^{5}+0.11233614271173\Phi^{6}-0.02219920555621\Phi^{7}+0.002974771630276\Phi^{8}-0.0002579193521337\Phi^{9}+0.000013046952630\Phi^{10}.$$

Using n=3 as the order of reaction, we obtain the left boundary derivatives, resulting

(21) $$X_{0}=\overset{˜}{z}(0),\;X_{1}=z^{\prime }(0)=0,$$

and

(22) $$X_{2}=\Phi^{2}{\left(X_{0}\right)}^{3},X_{3}=3\Phi^{2}X_{0}^{2}X_{1},X_{4}=3\Phi^{2}X_{0}(\Phi^{2}X_{0}^{4}+2X_{1}^{2})\vdots$$

Next, we obtain derivatives for the right boundary of (17), resulting

(23) $$Y_{0}=z(1)=1,\;Y_{1}=\overset{˜}{z^{\prime }}(1),$$

and

(24) $$Y_{2}=\Phi^{2}Y_{0}^{3},Y_{3}=3\Phi^{2}Y_{0}^{2}Y_{1},Y_{4}=3\Phi^{2}Y_{0}(2Y_{1}^{2}+\Phi^{2}Y_{0}^{4}),\vdots$$

**Figure 4:**
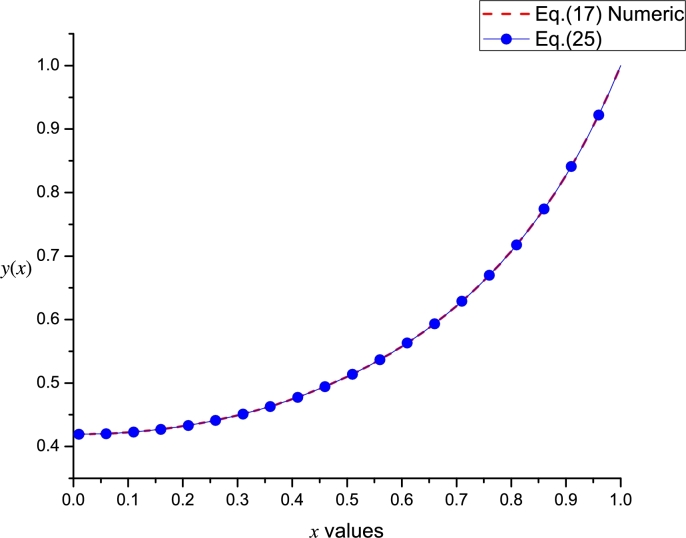
Comparison between numerical solution of (17) and (25).

Finally, as a particular case, we present the order [4,4] Leal-polynomial (B.5) for Φ=3 (see Fig. 4), resulting

(25) $$\overset{˜}{z}(x)=2.87603920279x^{9}-10.14322701918x^{8}+13.81278908889x^{7}-8.38517237774x^{6}+1.955966300497x^{5}+0.13165296608x^{4}+0.33237923463x^{2}+0.41957260404.$$

Now, we proceed to find the Taylor series ${\overset{˜}{z}}_{L}(x)$ (expanded at x=0) and ${\overset{˜}{z}}_{R}(x)$ (expanded at x=1) for (17) considering Φ=3 and n=3, resulting

(26) $${\overset{˜}{z}}_{L}(x)=0.419572604039+0.332379234630x^{2}+0.131652966078x^{4}+0.0625761239134x^{6}+0.0289169319377x^{8}+0.0133082058216x^{10},{\overset{˜}{z}}_{R}(x)=-1.08822786350+2.08822786350x+4.50{\left(x-1\right)}^{2}+9.39702538575{\left(x-1\right)}^{3}+19.9365651223{\left(x-1\right)}^{4}+42.1557095469{\left(x-1\right)}^{5}+89.1503602703{\left(x-1\right)}^{6}+188.522550760{\left(x-1\right)}^{7}+398.667271134{\left(x-1\right)}^{8}+843.056676863{\left(x-1\right)}^{9}+1782.80200925{\left(x-1\right)}^{10}.$$

**Figure 5:**
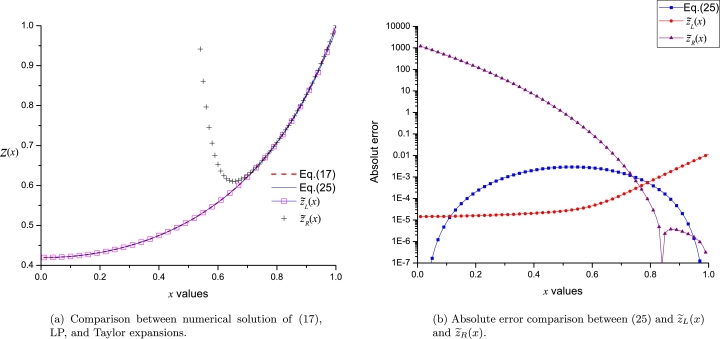
Comparing LP (25) and Taylor series expansions (26).

Equations (26) exhibit poor accuracy on the opposite frontier to their respective expansion points, on the contrary to LPs, that satisfy both frontiers (see Fig. 5). In addition, Fig. 5b shows the best accuracy of LP in comparison to ${\overset{˜}{z}}_{L}(x)$ and ${\overset{˜}{z}}_{R}(x)$. In general terms, the power of LP lies in its ability to expand, at the same time, on severals EPs, reducing the error in the inner region, whilst presenting high accuracy on the boundaries.

### 4.3 Model for chronic myelogenous leukemia (CML) and T cell interaction

Chronic myelogenous leukemia (CML) is a cancer that affects the blood system. The treatment of this disease is an important matter of public health. Therefore, [22] reported a mathematical model for the human immune system's response to CML, as

(27) $$C^{\prime }(t)=r_{c}C(t)\ln(\frac{C_{max}}{C(t)})+d_{c}C(t)+\gamma_{c}C(t)T_{e}(t),T_{e}^{\prime }(t)=\alpha_{n}k_{n}T_{n}(t)(\frac{C(t)}{C(t)+\eta })+\alpha_{e}T_{e}(t)(\frac{C(t)}{C(t)+\eta })+d_{e}T_{e}(t)+\gamma_{e}C(t)T_{e}(t),T_{n}^{\prime }(t)=s_{n}d_{n}T_{n}(t)-k_{n}T_{n}(t)(\frac{C(t)}{C(t)+\eta }),$$

where prime denotes differentiation with respect to *t*, C(t) is chronic myelogenous leukemia (CML) cancer cells, $T_{e}(t)$ is effector T(t) cells specific to chronic myelogenous leukemia (CML), and $T_{n}$ are naive T cells. $T_{n}$ consists of all naive T cells (specific and non-specific for CML). These cells either have not yet been exposed to professional antigen-presenting cell (APC), or else are not CML-specific. The $T_{e}(t)$ cells have differentiated from $T_{n}$ cells upon activation by a professional APC peptide-MHC (major histocompatibility complex) and costimulators.

Using the routine PLeal2 from Appendix C, we obtain $\overset{˜}{C}(t)$, $\overset{˜}{T_{e}}(t)$, and $\overset{˜}{T_{n}}(t)$, using [0,4,4], [1,4,3], and [1,4,3] as the order for expansions, respectively; Considering the expansion points [0,250,700] for $\overset{˜}{C}(t)$, [0,1/100,2/100] for $\overset{˜}{T_{e}}(t)$, and [0,10,30] for $\overset{˜}{T_{n}}(t)$. The numerical values employed as expansion points for LP are

(28) $$C(0)=10000,T_{e}(0)=20,T_{n}(0)=1510,C(250)=4419.385063710,T_{e}(250)=0.000038774,T_{n}(250)=1.305788914,C(700)=3481.304337010,T_{e}(700)=0.000047785,T_{n}(700)=1.314955484,$$

(29) $$C(0)=10000,T_{e}(0)=20,T_{n}(0)=1510,C(1/100)=9998.191123730,T_{e}(1/100)=0.088734514,T_{n}(1/100)=1505.663630490,C(2/100)=9997.567327780,T_{e}(2/100)=0.021592272,T_{n}(2/100)=1501.339729470,$$

and

(30) $$C(0)=10000,T_{e}(0)=20,T_{n}(0)=1510,C(10)=9418.491126270,T_{e}(10)=0.001297153,T_{n}(10)=86.218493411,C(30)=8442.583089090,T_{e}(30)=0.000025951,T_{n}(30)=1.559330316,$$

for $\overset{˜}{C}(t)$, $\overset{˜}{T_{e}}(t)$, and $\overset{˜}{T_{n}}(t)$, respectively. The initial conditions (t=0) and parameters (see Table 1) are the same as reported at [22]. We obtained the numerical points for expansions for CML model using the Fehlberg fourth-fifth order Runge-Kutta method with degree four interpolant (RKF45) [38], [39], integrated as an option of the build-in routine ‘dsolve’ from Maple 15.

**Table 1:** Parameters of CML as reported at [22].

Parameters	*s*_*n*_	*d*_*n*_	*d*_*e*_	*d*_*c*_	*k*_*n*_	*η*	*α*_*n*_	*α*_*e*_	*C*_*max*_	*r*_*c*_	*γ*_*e*_	*γ*_*c*_
Values	0.37	0.23	0.30	0.024	0.062	720	0.14	0.98	230000	0.0057	0.057	0.0034

Hence, using the Taylor series method for the rest of the unknown derivatives, it results the following LPs

(31) $$\overset{˜}{C}(t)=1.7957616199597328302\times 10^{-24}t^{10}-8.736323285667383102\times 10^{-21}t^{9}+1.88494630403252974554\times 10^{-17}t^{8}-2.38408789877582784352\times 10^{-14}t^{7}+1.97259991011900811406\times 10^{-11}t^{6}-1.13194230255477277500\times 10^{-8}t^{5}+0.468331318413133947316\times 10^{-5}t^{4}-0.144332241555328731232\times 10^{-2}t^{3}+0.340474709975452907497t^{2}-60.6609269707040807140t+10000,$$

(32) $$\overset{˜}{T_{e}}(t)=-2.4631153674227935057\times 10^{21}t^{11}+3.6907498886834987851\times 10^{20}t^{10}-2.49879762871539192964\times 10^{19}t^{9}+1.01286190380901325664\times 10^{18}t^{8}-2.74637685959409733618\times 10^{16}t^{7}+5.2724643113224999138\times 10^{14}t^{6}-7.3925602626498773326\times 10^{12}t^{5}+7.67525489379661437066\times 10^{10}t^{4}-5.875063044663287255826\times 10^{8}t^{3}+3.207290775010397048598\times 10^{6}t^{2}-11375.48992537313432836t+20,$$

and

(33) $$\overset{˜}{T_{n}}(t)=9.56607568808819127\times 10^{-15}t^{12}-2.456208760827606330\times 10^{-12}t^{11}+2.892616776470605857\times 10^{-10}t^{10}-2.082925162558036826\times 10^{-8}t^{9}+0.10336423245416374749\times 10^{-5}t^{8}-0.3784337676899099136\times 10^{-4}t^{7}+0.10685340804426625975\times 10^{-2}t^{6}-0.02390549881717902246t^{5}+0.42702990412118744927t^{4}-5.9856422248464062060t^{3}+62.4944059872809647654t^{2}-434.2620895522388059701t+1510.$$

**Figure 6:**
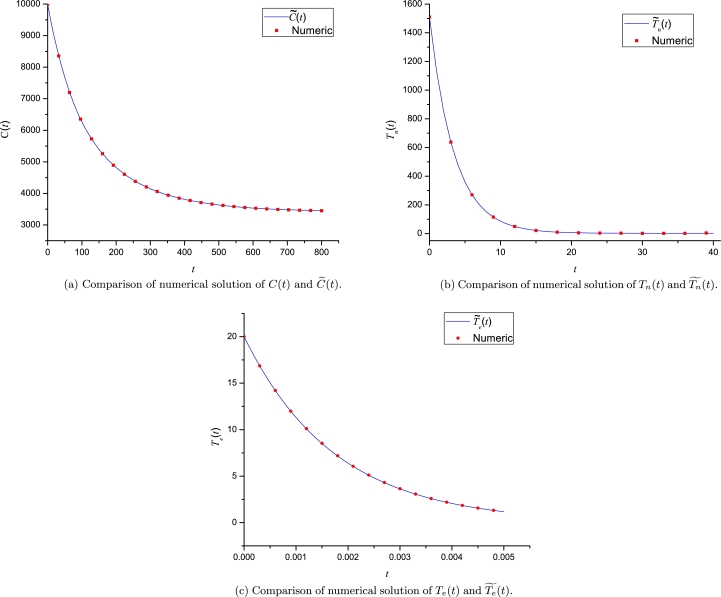
Numerical solutions and LP approximations for (27).

Fig. 6 illustrates a comparison among the numerical solution for (27) and the LP approximations, resulting in a wide domain of convergence. If users require a wider domain of convergence, they must select a higher order for LPs and a set of convenient expansion points.

### 4.4 Thomas-Fermi equation

The Thomas-Fermi (T-F) equation is a nonlinear singular differential equation defined on a semi-infinite domain [23], [24] as

(34) $$y^{\prime \prime }-\frac{1}{\sqrt{x}}y^{\frac{3}{2}}=0,\;x\in [0,\infty ],\;y(0)=1,\;y(\infty )=0,$$

where prime denotes differentiation with respect to *x*.

T-F equation is a starting point to understand nuclear physics. In fact, this model helps to study the potential and charge densities in [24]: atoms, molecules, atoms in strong magnetic fields, metals and crystals and dense plasmas, among others.

Using the routine PLeal2 from Appendix C, we obtain ${\overset{˜}{y}}_{1}(x)$ and ${\overset{˜}{y}}_{2}(x)$, using [0,12,12] and [4,10,10] as the order for expansions, respectively; Considering the expansion points [0,1,5] for ${\overset{˜}{y}}_{1}(x)$ and [5,10,25] for ${\overset{˜}{y}}_{2}(x)$. The numerical values [23] employed as expansions points for LP, are

(35) $$y(0)=1,y(1)=0.4240080520807056,y^{\prime }(1)=-0.2739890515933062,y(5)=0.07880777925136990,y^{\prime }(5)=-0.02356007495470051,$$

and

(36) $$y(5)=0.07880777925136990,y^{\prime }(5)=-0.02356007495470051,(10)=0.02431429298868086,y^{\prime }(10)=-0.004602881871269254,y(25)=0.003473754416765632,y^{\prime }(25)=-0.0003240429977697511,$$

for ${\overset{˜}{y}}_{1}(x)$ and ${\overset{˜}{y}}_{2}(x)$, respectively.

Hence, using the Taylor series method to obtain the rest of the unknown derivatives, we construct a piece-wise approximation

(37) $$\overset{˜}{y}(x)=\left\{ \begin{array}{cc} {\overset{˜}{y}}_{1}(x) & \;0\leq x<5,\\ {\overset{˜}{y}}_{2}(x) & \;5\leq x\leq 25. \end{array}\right.$$

**Figure 7:**
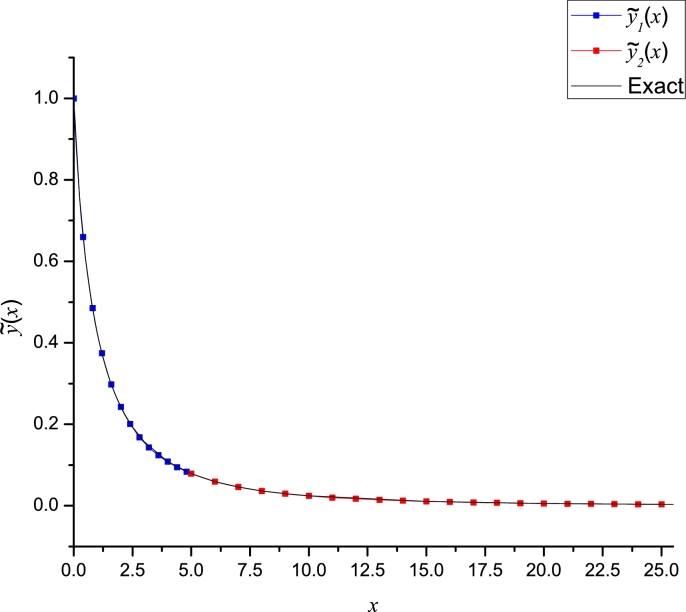
LP of TF and its numerical solution [23] (“exact”).

Fig. 7 shows the good agreement among the numerical solution [23] (considered as the exact solution for comparison purposes) and the LP approximation (37) (see Appendix D for the producing polynomials). In addition, Table 2 presents a comparison among the numerical solution, our scheme and other ones reported in literature. As we can note from such table, (37) presents the best accuracy regarding the other approximations. The work [40] reported an order one approximation using Laplace transform HPM, nonetheless, its accuracy is poor. Works [41] and [42] reported HAM approximations (asymptotic) of order 60 and 100, respectively, which produces too cumbersome demanding more computing resources to be evaluated than our proposal.

**Table 2:** Comparison between numerical solution of (34), Leal-Polynomials and other reported approximations.

*x*	Numerical [23]	(37)	[40]	[41]	[42]
0.25	0.755201465	0.755264750	0.708502932	0.776191000	0.755202000
0.50	0.606986383	0.606986568	0.570491745	0.615917000	0.606987000
0.75	0.502346846	0.502346846	0.485018011	0.505380000	0.502347000
1.00	0.424008052	0.424008052	0.421167227	0.423772000	0.424008000
1.25	0.363201414	0.363201414	0.369542163	0.362935000	0.363202000
1.50	0.314777464	0.314777456	0.326298526	0.314490000	0.314778000
1.75	0.275451328	0.275450737	0.289315779	0.275154000	0.275451000
2.00	0.243008507	0.242999650	0.257253699	0.242718000	0.243009000
2.25	0.215894627	0.215842348	0.229210094	0.215630000	0.215895000
2.50	0.192984123	0.192821354	0.204540381	0.192795000	0.192984000
3.00	0.156632673	0.156253373	0.163464474	0.156719000	0.156633000
4.00	0.108404257	0.108395165	0.105504623	0.109632000	0.108404000
5.00	0.078807779	0.078807779	0.068778618	0.081629600	0.078807800
6.00	0.059422949	0.059422949	-	0.063816200	0.059423000
7.00	0.046097819	0.046097819	-	0.051800500	0.046097800
8.00	0.036587255	0.036587255	-	0.043285900	0.036587300
9.00	0.029590935	0.029590935	-	0.037002300	0.029590900
10.0	0.024314293	0.024314293	-	0.032208100	0.024314300
15.0	0.010805359	0.010805355	-	0.019184300	0.010805400
20.0	0.005784941	0.005784920	-	0.013493700	0.005784940
25.0	0.003473754	0.003473754	-	0.010357000	0.003473750

### 4.5 High order nonlinear differential equations with discontinuities

In this section we will test the LPs to approximate nonlinear problems with discontinuities [25], [26], [27], [28], [29] by selecting the expansion points before and after the discontinuity constructing unified approximations. Then, let us to approximate this equation of order five

(38) $$s^{\left(5\right)}+5\text{sgn}(x)s+s^{2}+0.5x^{3}=0,s(0)=1,s^{\prime }(0)=0,s^{\prime \prime }(0)=-1,s^{\prime \prime \prime }(0)=0,s^{\left(4\right)}(0)=4,$$

where prime denotes differentiation with respect to *x*.

The discontinuity of (38) is produced by the abrupt change of sign at x=0 due to the term sgn [29] defined as

(39) $$\text{sgn}(x)=\left\{ \begin{array}{cc} -1 & \;x<0,\\ +1 & \;x\geq 0. \end{array}\right.$$

Using the routine PLeal from Appendix A, we obtain $\overset{˜}{s}(x)$ using order-sixth for both expansion points (EP); Considering a=−2 and b=2 as EP. The numerical values (RKF45 from Maple) employed to feed the LP, are

(40) $$y(-2)=0.648088150991336,y^{\prime }(-2)=-0.815309348835143,y^{\prime \prime }(-2)=2.02767227373873,y^{\prime \prime \prime }(-2)=-0.578006581278967,y^{\left(4\right)}(-2)=-3.91626570622947,y(2)=0.192417603031347,y^{\prime }(2)=-0.261385250609700,y^{\prime \prime }(2)=0.0744849115582207,y^{\prime \prime \prime }(2)=-1.80940087326309,y^{\left(4\right)}(2)=-5.07356458566883.$$

Resulting the following LP

(41) $$\overset{˜}{s}(x)=0.990105593717159-4.17064647197578\times 10^{-8}x^{13}+0.194973588901386\times 10^{-5}x^{12}+0.148555106327207\times 10^{-5}x^{11}-0.658336009356596\times 10^{-4}x^{10}-0.218312117642609\times 10^{-4}x^{9}+0.974779868270925\times 10^{-3}x^{8}+0.353102513477760\times 10^{-3}x^{7}-0.0127269758674467x^{6}-0.00824373881700278x^{5}+0.122310036350571x^{4}-0.109185884029433\times 10^{-3}x^{3}-0.475600749142705x^{2}+0.587852000888986\times 10^{-4}x$$

For comparison purposes, we will construct the Taylor expansions at x=2 and x=−2, resulting

(42) $${\overset{˜}{s}}_{R}(x)=0.1924176031-0.261385250609700(x-2)+0.0372424557791103{\left(x-2\right)}^{2}-0.301566812270829{\left(x-2\right)}^{3}-0.211398524419780{\left(x-2\right)}^{4}-0.0416592712399046{\left(x-2\right)}^{5}-0.637844930556523\times 10^{-2}{\left(x-2\right)}^{6}-0.129716933931645\times 10^{-2}{\left(x-2\right)}^{7}+0.170142387585177\times 10^{-3}{\left(x-2\right)}^{8}+0.647691125976229\times 10^{-4}{\left(x-2\right)}^{9}+0.450653357299183\times 10^{-5}{\left(x-2\right)}^{10}-0.112965154317250\times 10^{-5}{\left(x-2\right)}^{11}-0.127049656023453\times 10^{-5}{\left(x-2\right)}^{12}-4.59302355246024\times 10^{-7}{\left(x-2\right)}^{13},$$

and

(43) $${\overset{˜}{s}}_{L}(x)=0.6480881493-0.8153093488(x+2)+1.013836137{\left(x+2\right)}^{2}-0.09633443021{\left(x+2\right)}^{3}-0.1631777378{\left(x+2\right)}^{4}+0.05683685420{\left(x+2\right)}^{5}-0.01252744734{\left(x+2\right)}^{6}+0.002416801974e{\left(x+2\right)}^{7}+0.1185080976\times 10^{-3}{\left(x+2\right)}^{8}-0.1183419317\times 10^{-3}{\left(x+2\right)}^{9}+0.4621946773\times 10^{-5}{\left(x+2\right)}^{10}+0.6635469569\times 10^{-5}{\left(x+2\right)}^{11}-0.1664161023\times 10^{-5}{\left(x+2\right)}^{12}+9.133055591\times 10^{-8}{\left(x+2\right)}^{13},$$

respectively.

**Figure 8:**
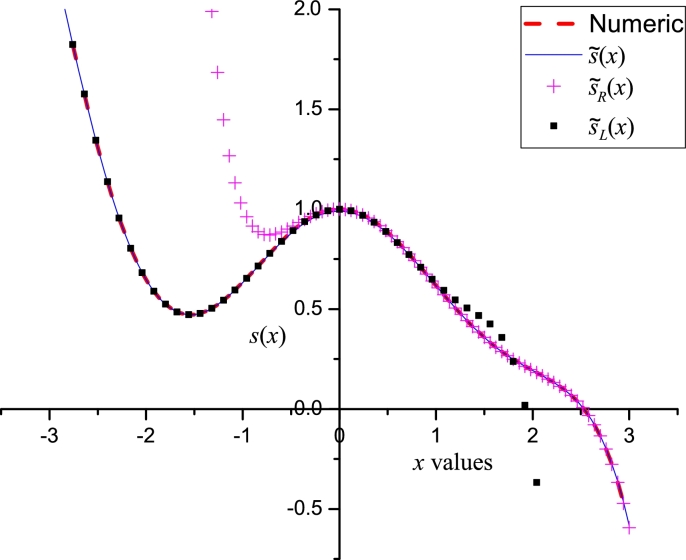
Comparison among the numerical solution of (38) and (41), (43) and (42).

Fig. 8 depicts the comparison among the numerical solution of (38), and its approximations (41), (43) and (42). The numerical solution was obtained solving (38) as two equations, one for x∈[−3,0] and the other one for x∈[0,3], using the RKF45 algorithm from Maple 15. It is important to note that the LP approximation exhibits a wide domain of convergence (x∈[−3,3]), whilst - as expected - $s_{L}(x)$ and $s_{R}(x)$ presents a good agreement for the domains: [−3,0] and [0,3], respectively; Because the discontinuity at x=0, both Taylor expansions are blind to what happens crossing the discontinuity. This issue will affect to any technique based on Taylor as classical perturbation method, homotopy perturbation method [40], homotopy analysis method [42], differential transform method [43], among others. Nonetheless, our proposal can deal with this issue, opening a wide scope of applications of LP to this kind of problems in physics.

## 5 Computing convergence and error

In this section we will analyse the error difference among the successive orders of LP to visualize the convergence of the approximation procedure as follows

(44) $$\text{convergence}=\sqrt{\frac{1}{b-a}\int_{a}^{b}{\left(L_{q+1}(x)-L_{q}(x)\right)}^{2}dx},$$

where *q* denotes the successive orders of the Leal-polynomials (*L*); *a* and *b* extreme EPs. Even more, we calculated the error between the numerical solution and different *q* orders of LP, using

(45) $$\text{error}=\sqrt{\frac{1}{b-a}\int_{a}^{b}{\left(H(x)-L_{q}(x)\right)}^{2}dx},$$

where H(x) represents the numerical solution considered as the exact solution for practical purposes.

**Figure 9:**
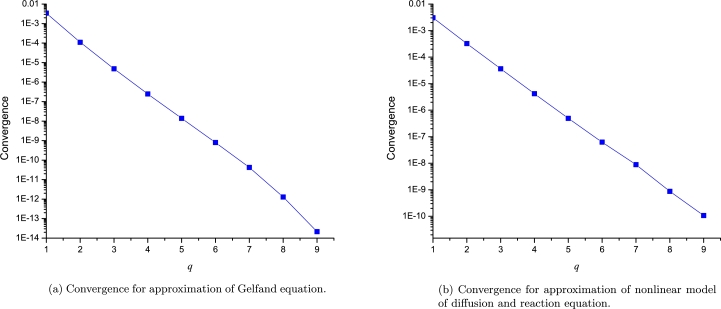
Computed convergence for both study cases.

**Figure 10:**
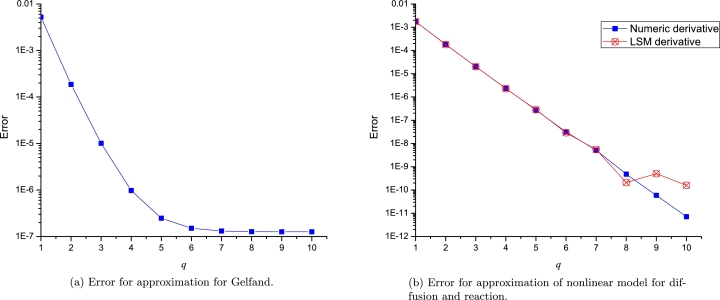
Error for approximations of both study cases.

On one hand, Fig. 9a and Fig. 9b show the convergence of different orders of LP to approximate (8) and (17), respectively. It is important to note that for both study cases, the error among the successive orders of LP describes a tendency for very low values (below $1\times 10^{-9}$). On the other hand, Fig. 10a and Fig. 10b depict the error between exact solution and 10 orders of LP, resulting a clear tendency of a decaying to zero. Although, Fig. 10b shows an unexpected error increasing at order q=9 due to the lack of accuracy of the LSM derivative; Nonetheless, we solved such issue by using the numerical derivative with 12 significant digits as depicted in the same figure. It is important to note that LPs with two expansion points of the same *q*-order, produces a polynomial of degree r=2q+1.

For the case of CML, we compute the convergence and error for the approximation of the three involved variables. As the three approximations are expanded at three points, we will perform the analysis using the following sequence of incremental orders:

(46) q=[[0,0,0],[0,0,1],[0,0,2],[0,0,3],[0,0,4],[0,1,4],[0,2,4],[0,3,4],[0,4,4]],

(47) q=[[1,0,0],[1,0,1],[1,0,2],[1,0,3],[1,1,3],[1,2,3],[1,3,3],[1,4,3],[1,5,3]],

and

**Figure 11:**
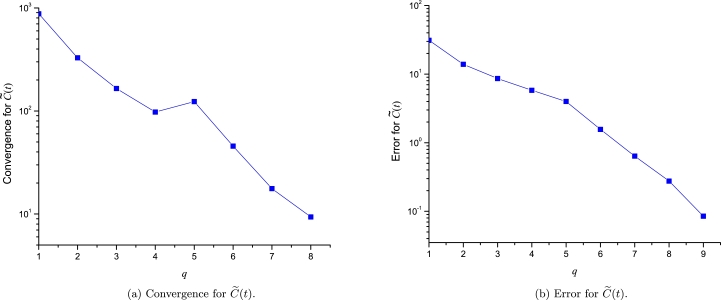
Convergence and error analysis for $\overset{˜}{C}(t)$.

**Figure 12:**
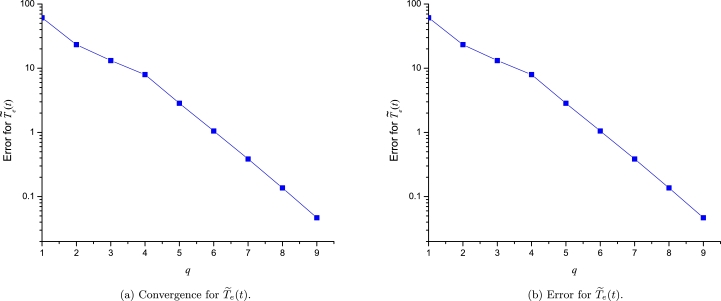
Convergence and error analysis for ${\overset{˜}{T}}_{e}(t)$.

**Figure 13:**
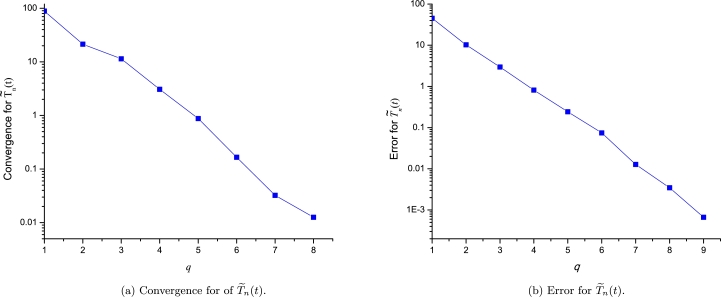
Convergence and error analysis for ${\overset{˜}{T}}_{n}(t)$.

(48) q=[[1,1,1],[1,1,2],[1,1,3],[1,2,3],[1,3,3],[1,4,3],[1,4,4],[1,4,5],[1,5,5]],

for $\overset{˜}{C}(t)$, ${\overset{˜}{T}}_{e}(t)$ and ${\overset{˜}{T}}_{n}(t)$, respectively. As expected, Figure 11, Figure 12, Figure 13 show a good convergence and error vanishing as the order of [$\overset{˜}{C}(t),{\overset{˜}{T}}_{e}(t),{\overset{˜}{T}}_{n}(t)$] approximations increases.

**Figure 14:**
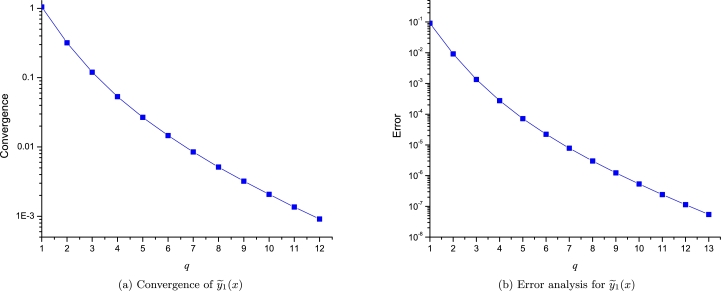
Convergence and error for ${\overset{˜}{y}}_{1}(x)$ of T-F equation.

We will compute the convergence and error for the approximation ${\overset{˜}{y}}_{1}(x)$ of T-F in the domain of x∈[0,5]; As it is expanded using three points, we will perform the analysis using the following sequence of incremental orders:

(49) q=[[0,0,0],[0,1,1],[0,2,2],[0,3,3],[0,4,4],[0,5,5],[0,6,6],[0,7,7],[0,8,8],[0,9,9],[0,10,10],[0,11,11],[0,12,12]],

resulting a clear convergence and error decaying to zero as the order increases as depicted in Fig. 14.

Finally, we will compute the convergence and error for the (41) for the problem with a discontinuity for the domain of x∈[−2,2]; As it is expanded using two points, we will perform the analysis using the following sequence of incremental orders:

(50) q=[[1,1],[2,2],[3,3],[4,4],[5,5],[6,6],[7,7],[8,8],[9,9]].

**Figure 15:**
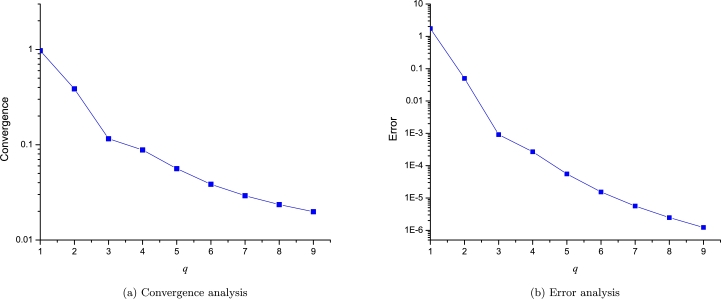
Convergence and error analysis for problem with a discontinuity (38).

Fig. 15 depicts how as the order of approximation increases the LP converge and an error decaying to zero. This result is interesting considering the discontinuity just in the middle of the EPs. This shows how LPs can be applied with success to this kind of problems.

## 6 Computing CPU time for Leal-polynomials

**Figure 16:**
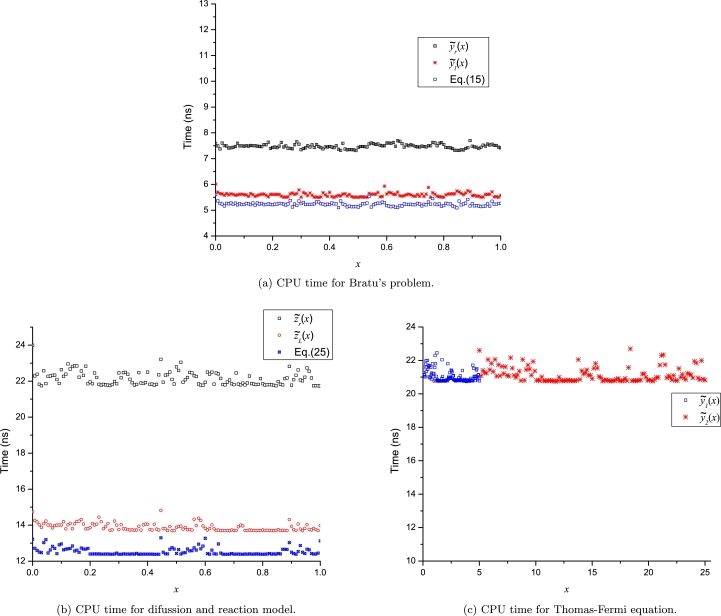
CPU times for LPs of: Bratu's problem, diffusion and reaction model and Thomas-Fermi equation.

**Figure 17:**
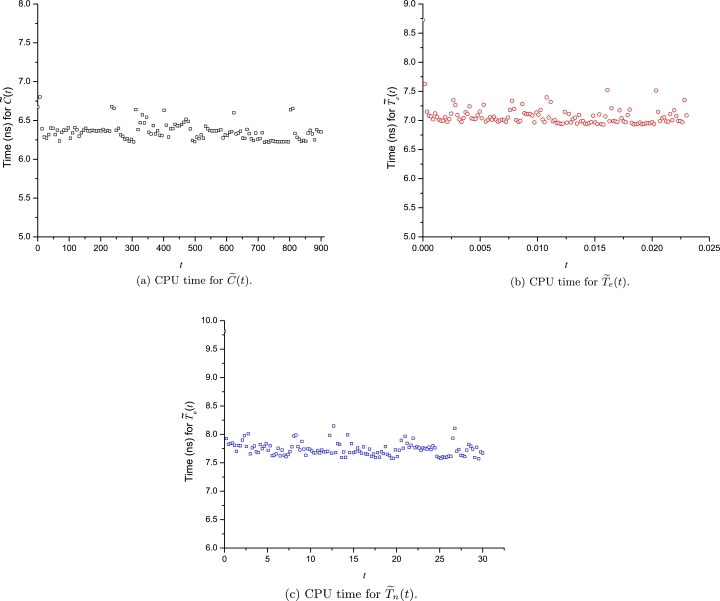
CPU time for LP-approximations of CML.

**Figure 18:**
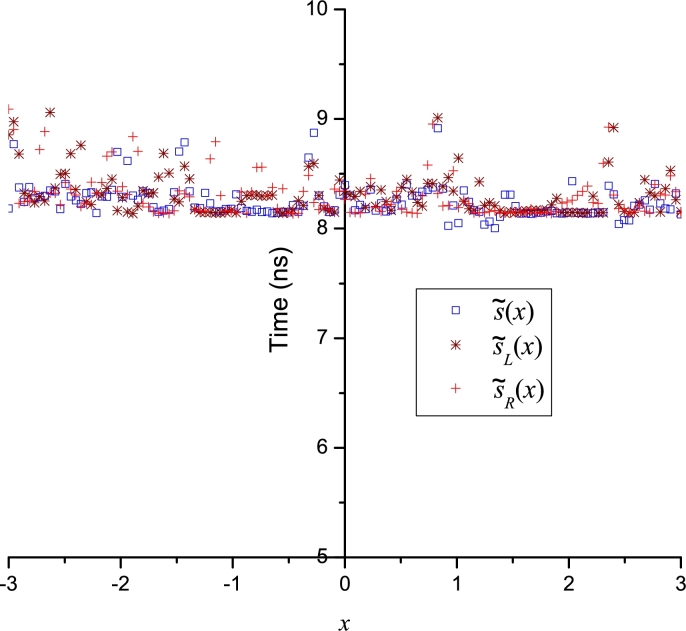
CPU time for LP approximation of the nonlinear problem with a discontinuity.

A computation time (CPU time) analysis for the LP approximations was achieved using Fortran 77/90, using as compiler the gfortran with level 3 optimization. The computer used was an Intel(R) Core(TM) i7-7700 CPU running at 3.60 GHz with 32 GB of main memory under Debian 10 (Linux). To circumvent the operative system fluctuations, the evaluation time was determined by the average time of 10 million calculations for each point (131 points). Figure 16, Figure 17, Figure 18 show how the LP approximations for all cases study require among 5 to 24 nanoseconds to be evaluated using real*8 data type (15 significant digits). Therefore, given the short computation times and the wide domain of convergence, we can conclude that the Leal-Polynomials are a powerful tool that can achieve: accurate, easy computable and compact approximations with a large domain of convergence. The Fortran code for the computation time of LPs for Thomas-Fermi equation is presented at Appendix D. The comparison of CPU time among LPs and Taylor series from Figs. 16a, 16b, and 18 show how the LPs exhibit lower or similar CPU times to the regular polynomials of Taylor series. Nonetheless, LPs exhibit a remarkable advantage of accuracy for the polynomials of the same degree as reported in Figs. 3b and 5b.

## 7 Discussion

**Figure 19:**
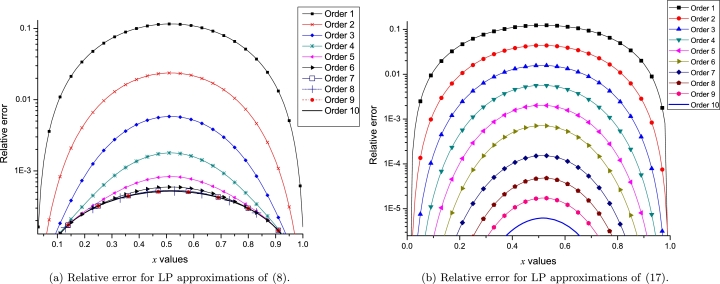
Relative error for different *q*-orders of LP. In fact, *q* means an order of [*q*,*q*].

The proposed scheme produces polynomials that satisfy the derivatives in multiple points at the same time as a mechanism to replicate the behaviour of the nonlinear problem. Figs. 19a and 19b present the relative error (RE) for orders 1 to 10 of LP, resulting a clear tendency of the RE to reduce its magnitude as the order of LP increases for (8) and (17) problems. This tendency of error reduction supports the above mentioned study of computational convergence and error analysis of LPs from Section 5. Also, CPU time consumption (see Section 6) of the proposed LPs shows that LPs can be used for intensive computing simulations.

The comparison among LPs and Taylor expansions from Figure 3, Figure 5, Figure 8 shows how the proposed polynomials exhibit a remarkable better accuracy for a wider domain. For the case of BVPs (8) and (17) LPs presented a reasonable accuracy for the region of [0,1], whilst, Taylor series exhibits a slow convergence; In addition, Taylor series will require an undetermined number (huge number) of terms to reach a similar error to the one presented for LPs (see section 5). In fact, the Taylor series expansion for CML model at t=0, shows a similar behaviour. For the case of a high order (38) equation with a discontinuity, we can conclude that LPs can be applied to the solution of this kind of problems, whilst, Taylor series cannot approximate this problem across the discontinuity point. Appendix A presents a Maple code to obtain symbolic Leal-polynomials of *q* order for the given *a* and *b* EPs. Appendix B presents specific examples of symbolic LP for orders [0,1,2,3,4]. Nevertheless, if the user requires more expansion points or different expansion orders for *a* and *b*, it is necessary to follow Algorithm 1, which is a straightforward task (see Appendix C).

We obtain the LP coefficients using a straightforward procedure that involves the solution of a system of linear equations (5), which makes LPs affordable in terms of their computational requirements to construct them; regarding the benefits in terms of accuracy, speed of evaluation and easy implementation. It requires future work to extend the power of LPs; among the open problems or challenges, we can mention:

1. Apply LP to approximate nonlinear differential equations of high-order >5. It is common to find in literature nonlinear differential equations of superior order for different applications, for instance, fluid mechanics, chemistry, biology, physics, among others. Therefore, approximating such problems using LP will challenge to prove their effectivity, in particular when stiffness or singularities are presented on nonlinear problems, which makes them difficult to solve even for numerical methods.
2. Apply the least square method (LSM) to general nonlinear models to obtain the function for unknown derivatives and not only polynomials (see (9), (10), (19), (20)). This line of research will produce some potential advantages: large domains of convergence for the derivatives, and more accurate derivatives which are useful - in particular - for high order LPs (see Fig. 10b).
3. It is frequent to deal with nonlinear problems exhibiting two or more intrinsic parameters. Therefore, extending the procedure to model unknown derivatives when two or more intrinsic parameters are presented is an important problem to solve. Here, we can apply different curve fitting techniques or algorithms, we can mention: multivariable nonlinear regression [44], neural networks [45], [46], piece-wise linear method [47], among many others. The required accuracy will determine the technique selection, how expensive is its evaluation in terms of speed (computational load), required memory, energy consumption, among others.
4. To change LSM is an attractive research line. For instance, to approximate derivatives, we can use some approximative methods as homotopy perturbation method [15], homotopy analysis method [14], Adomian decomposition method [48], [49], perturbation method [50], [51], among others. Therefore, such methods will provide semi-analytic derivatives with certain accuracy depending on the selected method and the problem to solve, but perhaps more compact, easy computable, and semi-analytic expressions than LSM does.
5. The power of LPs can be extended to approximate special or general functions (in terms of any combination of transcendental functions), like Taylor series does, but using multiple EPs. It is important to remark that to construct the LPs is required that special or general functions are continuous in the EPs.
6. In this work, we solved the Thomas-Fermi equation which is a singular problem that is hard to solve numerically or semi-analytically. However, LPs must be tested with other singular problems of different kind to prove its soundness.
7. LPs must be used to treat more nonlinear problems with discontinuities [25], [26], [27], [28], in particular real problems from physics.
8. The application of LPs to partial differential equations (PDEs) is an attractive line of research for future work because of the broad scope of applications of such equations. Multi-variable Taylor series must be the keystone for a future application of LPs to PDES.
9. As long as the numerical solution of a boundary-layer problem [52], [53], [54] is available, LPs can approximate such kind of problems.
10. Further research is required to apply LPs to approximate stiff nonlinear differential equations [55], [56], [57] or delay nonlinear differential equations [58], [59]; which arises in areas such as chemical engineering, nonlinear mechanics, biochemistry and life sciences.
11. Theoretical research work will be required to obtain a formal analysis of convergence; at least for particular nonlinear differential equations.
12. It is possible to propose a systematic multistage LP scheme by splitting the domain into segments that are easier to approximate as depicted for the study case of Thomas-Fermi equation.
13. Further work is required to implement a convergence-control scheme to increase the accuracy of LPs or its domain of convergence.

## 8 Conclusions

In this work we presented the Leal-polynomials (see Algorithm 1 for practical implementations) as an interesting and powerful tool to approximate nonlinear differential equations of different kind. The results of study cases show how our proposal produces accurate approximations. The main contribution of this method is its ability to produce multi-expansive polynomials, in contrast to Taylor series that is restricted to single point expansions. Such multi-expansions can produce accurate approximations in the inner region among the expansion points, whilst guaranteeing the satisfaction of the initial conditions or boundaries values of the problem. We also presented CPU computation analysis, computational convergence and error studies resulting: evaluation times in the order of nano-seconds, convergent polynomials and error reduction as the order of LP-approximations increases for all study cases. Furthermore, the proposed multi-expansive polynomials are straightforward to obtain (see Appendix A and Appendix C and easy to evaluate (see Appendix B). From the above-mentioned results, we can conclude that our proposal will have interesting applications for a wide scope of nonlinear differential equations emerging in science and engineering.

## Declarations

### Author contribution statement

H. Vazquez-Leal: Conceived and designed the experiments; Performed the experiments; Analyzed and interpreted the data; Contributed reagents, materials, analysis tools or data; Wrote the paper.

M. A. Sandoval-Hernandez: Conceived and designed the experiments; Performed the experiments; Analyzed and interpreted the data; Contributed reagents, materials, analysis tools or data.

U. Filobello-Nino, J. Huerta-Chua: Performed the experiments; Analyzed and interpreted the data; Contributed reagents, materials, analysis tools or data.

### Funding statement

This research did not receive any specific grant from funding agencies in the public, commercial, or not-for-profit sectors.

### Competing interest statement

The authors declare no conflict of interest.

### Additional information

Supplementary content related to this article has been published online at https://doi.org/10.1016/j.heliyon.2020.e03695.

No additional information is available for this paper.

## Supplementary material

The following Supplementary material is associated with this article:

Appendices - LealPoli.pdf Appendices. Appendices - LealPoli.pdf
