# Supplementary material for: The novel Leal-polynomials for the multi-expansive approximation of nonlinear differential equations
Source: Heliyon. 2020 Apr 14;6(4):e03695. doi: 10.1016/j.heliyon.2020.e03695 (PMC7160581; doi:10.1016/j.heliyon.2020.e03695)
Supplement: Appendices - LealPoli.pdf — Appendices. [file mmc1.pdf]

## Appendix A. Maple procedure to generate Leal-polynomials

```
#Procedure for Leal polynomials of q-Order
PLeal:= proc (qOrder::numeric, boundary_left::symbol, boundary_right::symbol)
local i, Ft, sys_a, sys_b, syst_solution, soluc, a, b, polinomL:
Ft:= add(c[i]*x^i, i=0..2*qOrder+1):
sys_a:= eval(Ft, x=a) =X[0], seq(eval(diff(Ft, x$i), x=a) =X[i], i=1..qOrder);
sys_b:= eval(Ft, x=b) =Y[0], seq(eval(diff(Ft, x$i), x=b) =Y[i], i=1..qOrder);
syst_solution:= [sys_a, sys_b]:
soluc:= solve(syst_solution, [seq(c[i], i=0..2*qOrder+1)]):
polinomL:= eval(Ft, soluc[1]):
return subs(a=boundary_left, b=boundary_right, polinomL):
end proc:
```

## Appendix B. First orders of Leal-polynomials

We present Leal-polynomials using PLeal$(q, 0, b)$ for $q = 0, 1, 2, 3, 4$ (See Appendix A). It is important to remember that the order $[q, q]$ of a LP means a polynomial degree of $2q + 1$.

$$\widetilde{y}_0(x) = -\frac{Y_0 - X_0}{b}x + X_0, \tag{B.1}$$

$$\begin{aligned}\widetilde{y}_1(x) = -\frac{1}{b^3}(-bX_1 - bY_1 - 2X_0 + 2Y_0)x^3 + \frac{1}{b^2}(-2bX_1 - bY_1 - \\ 3X_0 + 3Y_0)x^2 + X_1x + X_0,\end{aligned} \tag{B.2}$$

$$\begin{aligned}\widetilde{y}_2(x) = \frac{1}{2b^5}(-b^2X_2 + b^2Y_2 - 6bX_1 - 6bY_1 - 12X_0 + 12Y_0)x^5 - \\ \frac{1}{2b^4}(-3b^2X_2 + 2b^2Y_2 - 16bX_1 - 14bY_1 - 30X_0 + \\ 30Y_0)x^4 + \frac{1}{2b^3}(-3b^2X_2 + b^2Y_2 - 12bX_1 - 8bY_1 - \\ 20X_0 + 20Y_0)x^3 + \frac{1}{2}X_2x^2 + X_1x + X_0,\end{aligned} \tag{B.3}$$

$$\begin{aligned}\widetilde{y}_3(x) = \frac{1}{6b^7}(b^3X_3 + b^3Y_3 + 12b^2X_2 - 12b^2Y_2 + \\ 60bX_1 + 60bY_1 + 120X_0 - 120Y_0)x^7 - \\ \frac{1}{6b^6}(4b^3X_3 + 3b^3Y_3 + 45b^2X_2 - 39b^2Y_2 + \\ 216bX_1 + 204bY_1 + 420X_0 - 420Y_0)x^6 + \\ \frac{1}{2b^5}(2b^3X_3 + b^3Y_3 + 20b^2X_2 - 14b^2Y_2 + \\ 90bX_1 + 78bY_1 + 168X_0 - 168Y_0)x^5 - \\ \frac{1}{6b^4}(4b^3X_3 + b^3Y_3 + 30b^2X_2 - 15b^2Y_2 + \\ 120bX_1 + 90bY_1 + 210X_0 - 210Y_0)x^4 + \\ \frac{1}{6}X_3x^3 + \left(\frac{1}{2}\right)X_2x^2 + X_1x + X_0,\end{aligned} \tag{B.4}$$

$$
\begin{aligned}
\widetilde{y}_4(x) = \frac{1}{24b^9}(&-b^4X_4 + b^4Y_4 - 20b^3X_3 - 20b^3Y_3 - 180b^2X_2 + 180b^2Y_2 - \\
&840bX_1 - 840bY_1 - 1680X_0 + 1680Y_0)x^9 - \\
\frac{1}{24b^8}(&-5b^4X_4 + 4b^4Y_4 - 96b^3X_3 - 84b^3Y_3 - 840b^2X_2 + 780b^2Y_2 - \\
&3840bX_1 - 3720bY_1 - 7560X_0 + 7560Y_0)x^8 + \\
\frac{1}{12b^7}(&-5b^4X_4 + 3b^4Y_4 - 90b^3X_3 - 66b^3Y_3 - 756b^2X_2 + 636b^2Y_2 - \\
&3360bX_1 - 3120bY_1 - 6480bX_0 + 6480bY_0)x^7 - \\
\frac{1}{12b^6}(&-5b^4X_4 + 2b^4Y_4 - 80b^3X_3 - 46b^3Y_3 - 630b^2X_2 + 462b^2Y_2 - \\
&2688bX_1 - 2352bY_1 - 5040X_0 + 5040Y_0)x^6 + \\
\frac{1}{24b^5}(&-5b^4X_4 + b^4Y_4 - 60b^3X_3 - 24b^3Y_3 - 420b^2X_2 + 252b^2Y_2 - \\
&1680bX_1 - 1344bY_1 - 3024X_0 + 3024Y_0)x^5 + \frac{1}{24}X_4x^4 + \\
&\frac{1}{6}X_3x^3 + \frac{1}{2}X_2x^2 + X_1x + X_0.
\end{aligned} \tag{B.5}
$$

## Appendix C. Maple procedure to generate Leal-polynomials with three expansion points

This routine is called using this nomenclature $\mathsf{Pleal2}(q_a, q_b, q_c, a, b, c)$, where $[q_a, q_b, q_c]$ means the order of EPs $[a, b, c]$. This routine returns a polynomial in terms of $x$ as independent variable and $X[i]$ $(i = 0, 1, 2, \ldots, q_a)$, $Y[j]$ $(j = 0, 1, 2, , \ldots, q_b)$, and $Z[k]$ $(k = 0, 1, 2, , \ldots, q_c)$ as the successive derivatives for the three expansion points $[a, b, c]$. The degree of a LP with three expansion points is $r = q_a + q_b + q_c + 2$.

```
#Procedure for Leal polynomials of 3 expansion points and different orders of expansion
PLeal2 := proc (Order1::numeric, Order2::numeric, Order3::numeric,
a1::numeric, b1::numeric, c1::numeric)
local i, Ft, sys_a, sys_b, sys_c, syst_solution, soluc, a, b, polinomL, syst_solution1, c;
a := a1; b := b1; c := c1;
Ft := add(c[i]*x^i, i = 0 .. Order1+Order2+Order3+2);
sys_a := eval(Ft, x = a) = X[0], seq(eval(diff(Ft, x$i), x = a) = X[i], i = 1 .. Order1);
sys_b := eval(Ft, x = b) = Y[0], seq(eval(diff(Ft, x$i), x = b) = Y[i], i = 1 .. Order2);
sys_c := eval(Ft, x = c) = Z[0], seq(eval(diff(Ft, x$i), x = c) = Z[i], i = 1 .. Order3);
syst_solution := [sys_a, sys_b, sys_c];
soluc := solve(syst_solution, [seq(c[i], i = 0 .. Order1+Order2+Order3+2)]);
polinomL := eval(Ft, soluc[1]);
return polinomL;
end proc:
```

## Appendix D. CPU time analysis for LPs of T-F equation

```
!===================================================================
!!!!!!!!!!!!!!!!!!!!!!!!!!!!!!!!!!!!!!!!!!!!!!!!!!!!!!!!!!!!!!!!!!!!!!!!!!!!!!
program FermiCPUtime
!
!   Computation time: this code generates a table of CPU time for Thomas-Fermi Equation
!   Written by: Mario-A. Sandoval-Hernandez and Hector Vazquez-Leal

real :: start, finish, tt
integer jend,j
integer*8 i,maxiter
real*8 z,x,wmax,wmin,dw

maxiter=10000000
jend=130
wmin=0.0d0
wmax=5.0d0
dw=(wmax-wmin)/dble(jend)

!This code is to measure first LP for 0-5
OPEN (unit=20, File ='PLN1-CPUtime.txt', status ='unknown')
do j=0,jend
    x=wmin+dw*dble(j)
    call cpu_time(start)
    do i=0,maxiter
          z = 0.1D1 + (-0.1390288388182664127316459814070448lD1 + (0.2722904161472977775596254811255709 5D1 + &
          (-0.7268862478776735455672464558047201 5D1 + (0.1871694424854588953299995678474048 5D2 + &
          (-0.4047483102951418146932643470932198 9D2 + (0.7148883429485878836744255734622899 7D2 + &
          (-0.1032188836837586387002598850990456 2D3 + (0.1226394023464316526951175018368525 3D3 + &
          (-0.1207235935646123566362784140635828 6D3 + (0.9900505690418652896324845331840341D2 + &
          (-0.6792165892702254632602320510986002D2 + (0.3908613422927876037012338159465812D2 + &
          (-0.1889364299660239857052132260604519 7D2 + (0.7672973201174935144954084484236453D1 + &
          (-0.2615151481317086400369001268378197D1 + (0.7461923437404641650323622721987555D0 + &
          (-0.1775282936410760022035968163571131D0 + (0.3500506097880814132956559384157168D-1 + &
          (-0.5671605601983412948605265523251381D-2 + (0.7459866545323461169455446561308016D-3 + &
          (-0.7829742815376064508575493417115258D-4 + (0.6396427994316598057166921594507354D-5 + &
          (-0.3916610529D-6 + (0.1690045733D-7 + (-0.4580738704D-9 + &
          0.5864043695D-11 * x) * x) * x) * x) * x) * x) * x) * x) * x) * x) * x) * x) * x) * x) *&
          x) * x) * x) * x) * x) * x) * x) * x) * x) * x) * x) * x
     enddo
     call cpu_time(finish)
     tt=((finish-start)/maxiter)*1000000000
     write(20,"(0pf30.15,0pf25.15,0PF15.5)") x,z,tt
enddo
close (20)

!This code is to measure second LP for 5-25
OPEN (unit=20, File ='PLN2-CPUtime.txt', status ='unknown')
wmin=5.0d0
wmax=25.0d0
do j=0,jend
    x=wmin+dw*dble(j)
    call cpu_time(start)
    do i=0,maxiter
          z = 0.8725868091520197212015866719328 40D0 + (-0.7372643390255107588568221229763 09D0 + &
             (0.4226476292002553850251588435501 29D0 + (-0.1890024801428465798338142915988 56D0 + &
             (0.6800730502660971533083940065504 9D-1 + (-0.1998977170706170607802343314935 21D-1 +&
             (0.4856433768878234122755757807881 1D-2 + (-0.9848426697365854670555410668305 5D-3 + &
             (0.1680666326115480510463805472414 98D-3 + (-0.2429185717053715506042117952174 1D-4 + &
             (0.2988330731005067611326045203160 3D-5 + (-0.3139696411D-6 + (0.2823320742D-7 + &
             (-0.2174767070D-8 + (0.1434356232D-9 + (-0.8085848337D-11 + (0.3883426585D-12 + &
             (-0.1581202388D-13 + (0.5420148694D-15 + (-0.1549215473D-16 + (0.3643962945D-18 + &
             (-0.6926017683D-20 + (0.1036513284D-21 + (-0.1174963058D-23 + (0.9476588581D-26 + &
             (-0.4842893016D-28 + 0.1178143043D-30 * x) * x) * x) * x) * x) * x) * x) * x) * x) *&
              x) * x) * x) * x) * x) * x) * x) * x) * x) * x) * x) * x) * x) * x) * x) * x) * x
     enddo
     call cpu_time(finish)
     tt=((finish-start)/maxiter)*1000000000
```

```
     write(20,"(0pf30.15,0pf25.15,0PF15.5)") x,z,tt
enddo
close (20)
end program FermiCPUtime
```
